# Efficacy of a Six-Week-Long Therapist-Guided Online Therapy Versus Self-help Internet-Based Therapy for COVID-19–Induced Anxiety and Depression: Open-label, Pragmatic, Randomized Controlled Trial

**DOI:** 10.2196/26683

**Published:** 2021-02-12

**Authors:** Mohammed Al-Alawi, Roopa K McCall, Alya Sultan, Naser Al Balushi, Tamadhir Al-Mahrouqi, Abdullah Al Ghailani, Hilal Al Sabti, Abdullah Al-Maniri, Sathiya M Panchatcharam, Hamed Al Sinawi

**Affiliations:** 1 Department of Behavioral Medicine Sultan Qaboos University Hospital Muscat Oman; 2 Al Harub Medical Center Muscat Oman; 3 Euonia Clinic Muscat Oman; 4 Oman Medical Specialty Board Muscat Oman

**Keywords:** COVID-19, depression, anxiety, Oman, online therapy, randomized controlled trial, telehealth, therapy, mental health, e-mental health, self-help, distress

## Abstract

**Background:**

The COVID-19 pandemic has led to a notable increase in psychological distress, globally. Oman is no exception to this, with several studies indicating high levels of anxiety and depression among the Omani public. There is a need for adaptive and effective interventions that aim to improve the elevated levels of psychological distress due to the COVID-19 pandemic.

**Objective:**

This study aimed to comparatively assess the efficacy of therapist-guided online therapy with that of self-help, internet-based therapy focusing on COVID-19–induced symptoms of anxiety and depression among individuals living in Oman during the COVID-19 pandemic.

**Methods:**

This was a 6-week-long pragmatic randomized controlled trial involving 60 participants who were recruited from a study sample surveyed for symptoms of anxiety or depression among the Omani public amid the COVID-19 pandemic. Participants in the intervention group were allocated to receive 1 online session per week for 6 weeks from certified psychotherapists in Oman; these sessions were conducted in Arabic or English. The psychotherapists utilized cognitive behavioral therapy and acceptance and commitment therapy interventions. Participants in the control group received an automatic weekly newsletter via email containing self-help information and tips to cope with distress associated with COVID-19. The information mainly consisted of behavioral tips revolving around the principles of cognitive behavioral therapy and acceptance and commitment therapy. The primary outcome was measured by comparing the change in the mean scores of Patient Health Questionnaire-9 (PHQ-9) and General Anxiety Disorder-7 (GAD-7) scale from the baseline to the end of the study (ie, after 6 sessions) between the two groups. The secondary outcome was to compare the proportions of participants with depression and anxiety between the two groups.

**Results:**

Data from 46 participants were analyzed (intervention group n=22, control group n=24). There was no statistical difference in the baseline characteristics between both groups. Analysis of covariance indicated a significant reduction in the GAD-7 scores (*F*_1,43_=7.307; *P*=.01) between the two groups after adjusting for baseline scores. GAD-7 scores of participants in the intervention group were considerably more reduced than those of participants in the control group (β=−3.27; *P*=.01). Moreover, a greater reduction in mean PHQ-9 scores was observed among participants in the intervention group (*F*_1,43_=8.298; *P*=.006) than those in the control group (β=−4.311; *P*=.006). Although the levels of anxiety and depression reduced in both study groups, the reduction was higher in the intervention group (*P=*.049) than in the control group (*P=*.02).

**Conclusions:**

This study provides preliminary evidence to support the efficacy of online therapy for improving the symptoms of anxiety and depression during the COVID-19 crisis in Oman. Therapist-guided online therapy was found to be superior to self-help, internet-based therapy; however, both therapies could be considered as viable options.

**Trial Registration:**

ClinicalTrials.gov NCT04378257; https://clinicaltrials.gov/ct2/show/NCT04378257

## Introduction

### Background

In order to contain and minimize the impact of the COVID-19 pandemic, countries across the world have resorted to physical distancing, quarantining, and social isolation. This has remodeled health care services through the implementation of web-based and/or remote treatment [1,2]. This transition has also been applied to mental health care services, wherein it has been highly recommended owing to the expected rise in psychological distress during the pandemic [3]. The rise in the use of web-based services catering to patients’ psychological well-being has also been observed in Oman, where these services are generally provided in government hospitals or in the private sector. However, there is a dearth of literature regarding the efficacy of this treatment model globally, including in Oman.

On March 11, 2020, the World Health Organization declared COVID-19 a pandemic. More literature is now available detailing the impact of the pandemic on the mental health of people, even among those who are not infected or less at risk [4]. As a result of the pandemic, all members of society are now considered to be “vulnerable” to the infection. As such, there is now an increase in ethical practice education regarding online therapy services, despite it being available for the last two decades. Mental health practitioners were previously resistant to implementing an online or remote-based system as a lot of emphasis has been placed in the value of face-to-face health care practice [5]. However, contrary to this popular belief, online psychotherapy or e-therapy guided by a psychotherapist has shown favorable outcomes, particularly in cases of anxiety or clinical depression [6,7]. In fact, there is a growing need for guided online therapy, particularly catering to populations in regions where access to treatment facilities for mental health care is limited [2,8].

Conversely, online therapy can also be inaccessible to large populations, owing to insufficient network connectivity, lack of economic feasibility, personal and social stigma, and the lack of availability or awareness of accessing such facilities. Considering these factors, an increasing number of self-help applications, including mobile apps and websites, now provide users with free, basic mental and physical health care remedies and strategies to cope with psychological distress due to the COVID-19 pandemic. Previous studies have suggested that the outcomes of face-to-face interventions (ie, in-person or online) and self-help online resources have yielded effective results [9,10]. However, these trends are yet to be explored in Oman and in the Middle East, in general.

Studies in Oman have suggested that people are at a high risk of experiencing psychological distress during the COVID-19 pandemic [11]. Marital status, gender, pre-existing health conditions and financial status have been found to play a significant role in enhancing the risk of developing psychological distress during this ongoing health crisis [11]. On April 10, 2020, the Omani government issued a lockdown in order to manage the COVID-19 outbreak in the country. At the time of writing this manuscript, 7770 confirmed COVID-19 cases were reported in Oman [12]. Mental health sectors across Oman have launched various self-help and online services to provide health care from a distance. It is of value to understand the efficacy of these services in enhancing the mental health status in order to assist the Omani health authorities to provide focused and high-quality mental health services to vulnerable populations. Additionally, such tools, if well-developed, would have far-reaching potential, particularly for those in Oman who are unable to access mental health services.

### Hypothesis

We hypothesized that compared to internet-based (email-delivered) self-help therapy, therapist-guided online therapy is more efficient in reducing COVID-19–induced symptoms of anxiety and depression among individuals in Oman during the COVID-19 pandemic.

### Objective

This study aimed to investigate and comparatively assess the efficacy of a 6-week-long therapist-guided online therapy course with that of internet-based (email-delivered), self-help therapy focusing on COVID-19–related symptoms of anxiety and depression among individuals in Oman during the COVID-19 pandemic.

The findings of this study can assist mental health practitioners in Oman to effectively provide their services in a remote-based format to vulnerable populations and also provide policy makers with insights into the usefulness of mental health aids delivered online during the COVID-19 pandemic, and any potential future health crises, in Oman.

## Methods

### Study Design and Location

In this 6-week, open-label, comparative trial, participants were randomized to receive either therapist-guided online therapy or internet-based self-help therapy, using a fixed randomization schedule that allocated participants to the two treatment arms in a 1:1 ratio. This study was conducted virtually, using a secure encrypted video conference platform to deliver the online therapy to participants in the intervention arm who were located across Oman.

### Participant Allocation and Randomization

A software randomizer generated block randomization sequence (block size is 6) in a 1:1 ratio to balance the number of the participants in each study arm. Participant allocation to either the intervention or control arm was concealed from the study participants and researchers before the trial was initiated to avoid selection bias. This was done through a centralized service at the Department of Behavioral Medicine, Sultan Qaboos University, Muscat, Oman. Each coded, sealed, opaque envelope containing the participant’s treatment allocation was opened by research personnel not involved in the study or in the process of data collection. All participants had a code number allocated to them. As this was an open-label trial, the participant and the therapist who conducted the therapy were aware of the intervention status after the randomization process was completed. However, the outcome assessor, or the person who received the outcome assessment emails, was blinded to the participant’s allocation.

### Sample Size

The sample size was calculated using nMaster 2.0 software (Department of Biostatistics, CMC, Vellore). The Superiority hypothesis parallel clinical trial model was adopted to calculate the required sample size in each study arm to achieve a mean effect size of Glass’ Δ=0.75, as previously described [13]. Considering the power as 80% and a 5% rate of type-I error, the required sample size was 30 participants for each arm, after considering a 20% attrition rate.

### Participant Selection and Eligibility

Participants were screened for specific inclusion and exclusion criteria before they were enrolled into this study (Textbox 1). This study followed the guidelines outlined in the CONSORT (Consolidated Standards of Reporting Trials) checklist (Multimedia Appendix 1).

**Study inclusion and exclusion criteria.**

**Inclusion criteria**

- All Omanis and non-Omanis living in Oman
- Male or female
- Aged 18-65 years
- Patient Health Questionnaire-9 total scores ≥12 or Generalized Anxiety Disorder-7 total scores ≥10
- Access to the internet and video conferencing
- Able to participate in the trial and adhere to the trial protocol
- Provided written informed consent to participate in the trial

**Exclusion criteria**

- Pre-existing mental health disorders
- Diagnosis of moderate-to-severe intellectual disability
- Presence of alcohol or other substance use disorders (except for nicotine or caffeine)
- Suicidal or homicidal ideation at the baseline

### Intervention Arm: Therapist-Guided Online Therapy

Participants allocated to the intervention arm received weekly sessions from a trained and licensed psychologist in Oman via Zoom Video Conferencing platform (Zoom Video Communications, Inc.). The therapist sent an e-invitation URL to the participant via email prior to the scheduled meeting. The URL directed the participant to a screen where they would be able to see and interact with their therapist. The sessions were conducted once a week in either Arabic or English, as per the participant’s preference.

The initial sessions focused on building a rapport and providing psychological first aid. The following sessions employed principles of cognitive behavioral therapy (CBT) and acceptance and commitment therapy (ACT) interventions based on the therapist’s training background and what was determined as the best fit for the participant. Ongoing supervision was provided in 1-hour weekly online group meetings with other therapists, as well individual sessions with each therapist, if required. No treatment manuals were used; instead, therapists were asked to use therapy models for which they have received training. The following skills were determined to be relevant for these sessions: active listening, empathy, providing focus and structure, goal setting, and providing feedback. Behavioral interventions were considered applicable to treatment, including behavior activation, exposure, homework, and skills training. Components designated as unique to CBT such as discussion of automatic thoughts, core beliefs, and schemas; identification of cognitive distortions; and cognitive restructuring were also applied. Unique ACT components such as experiential acceptance and willingness, de-fusion, mindfulness training, and encouragement of value-driven living, were also applied by therapists while ensuring adherence to the same orientation throughout the sessions.

The final sessions primarily focused on relapse prevention and terminating the therapeutic relationship. After 6 sessions, participants received an email to complete the postintervention outcome assessment.

### Control Arm

Participants allocated to the control arm received an automatic weekly newsletter via email, containing self-help information and tips to cope with distress associated with COVID-19. The information mainly consisted of behavioral tips based on principles of CBT and ACT, such as focusing on positive cognitive reinforcement, strengthening relationships, and mindfulness practices. Participants were requested to use this information to manage any distress that they might experience. After 6 weeks, participants in the control group were assessed for their mood and anxiety symptoms via email-based questionnaires.

### Outcome Measures

Patient Health Questionnaire-9 (PHQ-9) is a self-administered measure used to make a tentative diagnosis of depression and monitor its severity. PHQ-9 has been validated in a number of studies involving different populations [14-16]. Al-Ghafri et al [17] examined the applicability and psychometric characteristics of the PHQ-9 among an Omani sample. A cutoff score of 12 gave the best trade-off between sensitivity (80.6%) and specificity (94%). Therefore, in this study, a cutoff score of 12 was used to indicate the presence of significant depression.

General Anxiety Disorder-7 (GAD-7) scale consists of a self-reported, 7-item questionnaire that allows for the rapid detection of generalized anxiety disorder [18]. Participants were asked if they were bothered by anxiety-related problems over the past 2 weeks by 7 questions on the GAD-7 scale, evaluated on a 4-point scale. The total scores ranged from 0 to 21. At a cutoff score of 9 based on the GAD-7 yielded a sensitivity of 89% and a specificity of 82% for detecting anxiety compared with a structured psychiatric interview [18]. However, the validation of an Arabic version of the GAD-7 scale indicated that a cutoff score of 10 had the best trade-off between sensitivity and specificity [19,20]. Therefore, in this study, we considered a total score of 10 and above as the cutoff for significant anxiety.

Data related to participants’ age, gender, marital status, number of children, highest qualification, studying abroad, occupation, working in health care, financial strains, physical health problems, mental health problems, self-quarantine, coping with illicit drugs, and email addresses were also collected.

The primary endpoint was to compare the differences in the mean scores of PHQ-9 and GAD-7 from the baseline to the end of the study (ie, after 6 sessions) between participants in the intervention and the control arms. The secondary endpoint was to compare the proportion of participants with significant psychological distress (ie, PHQ-9 total score ≥12 or GAD-7 total score ≥10) between the two study arms.

### Recruitment and Consent Procedure

Recruitment of participants took place between April 14 and May 30, 2020. Participants were recruited from a list of online survey respondents based in Oman with significant psychological distress during the COVID-19 pandemic [4]. This survey was conducted by the current trial team during the first two weeks of April 2020 and included 1539 respondents from different regions of Oman. The prevalence of psychological distress among the trial sample was 30%. The research assistant contacted eligible participants by email, and interested participants were briefed about the trial protocol. Potential participants received a detailed explanation of the objectives, procedures, and risks of the trial protocol. Based on the autonomy principle, the subject had the right to decline to participate or to withdraw from the study at any time without prejudice. After the consent form was read and discussed with their family, if participants wished to get their family’s opinion, the participants signed an electronic informed consent to participate in this study. All interaction with the study participants, including explanation and consent process, was conducted over private telephonic or video interviews.

### Follow-up Visits and Assessment Procedures and Data Management

After baseline assessment and receiving 6 sessions of therapist-guided online therapy or self-help therapy, the study participants were assessed for outcome measures at week 6. For this, participants were sent email links to the questionnaires (GAD-7 and PHQ-9). Over the first 6 weeks, each remote therapy session included checking the consent, compliance with protocol, and whether any adverse effects such as worsening of anxiety or depression symptoms persisted; this was carried out for participants in both the intervention and control arms. In the self-help (control) arm, we asked the participants, via weekly emails, to confirm receipt of the therapy material via an email to the research assistant. Additionally, we asked them to report any adverse events by contacting the research assistant through a phone number provided to them at the beginning of the study.

Each participant’s data were assigned a unique code (serial number). All data collected were initially recorded in a specified file for each participant for every session completed and then transferred to the EpiData sheet at the end of all remote sessions.

### Statistical Analyses

The data were double-entered into an electronic database EpiData (V.2.2, EpiData Association, 2000-2008) to ensure accuracy and then exported to SPSS software (version 20.0; IBM Corp.). Continuous variables were summarized as means and SD, and categorical variables were presented as frequencies. Analysis of covariance was used in examining pre- and postintervention differences in the mean scores of PHQ-9 and GAD-7 between the intervention and control arms while considering the influence of uncontrolled independent variables, such as preintervention scores. Categorical variables and proportions of participants with anxiety or depression were investigated using the chi-square test or Fisher exact test, when appropriate. Significance level was set at *P<*.05.

### Ethics Approval

This study followed the guidelines of the Declaration of Helsinki, 2001 [21]. Participation was voluntary; each participant had the right to withdraw from the trial at any time for any reason, and their withdrawal did not affect them in any way. This study was granted ethical approval by the research ethics committee at the College of Medicine and Health Science, Sultan Qaboos University, Muscat, Oman (MREC#2103). This trial was registered at ClinicalTrials.gov, registration number NCT04378257.

### Data Availability Statement

Data are available upon reasonable request directed to the corresponding author (MA).

## Results

Data related to 46 participants were analyzed in this trial (Table 1). Of the 46 participants, 22 (48%) were allocated to the intervention arm and 24 (52%) were allocated to the control arm. The mean age of the participants was 28.51 (SD 8.70) years, and approximately 78% (36/46) of them were female. Most participants (26/46, 57%) were single and about one-third had children (15/46, 33%). Furthermore, most participants (34/46, 74%) had completed education up to the college level, and 33% (15/46) of the participants were working in the health care industry. Financial instability was reported by 28% (13/46) of the study participants. The majority (30/46, 65%) of the participants were self-quarantining because of the COVID-19 pandemic. The difference in baseline scores and proportions of anxiety and depression among participants in the two arms were not statistically significant. Additionally, there was no statistically significant differences in the baseline characteristics of participants in the two arms. Attrition rate was 26% for the intervention arm and 20% for the control arm. Overall, the characteristics of participants who were lost to follow-up did not differ from the characteristics of the remainder of the participants. Regarding adverse events, 1 participant who was allocated to the intervention arm reported safety concerns before starting the therapy. The participant was immediately referred for a psychiatric assessment at Sultan Qaboos University Hospital.

A univariate analysis of covariance was conducted to compare the effectiveness of the intervention between the two study arms, while adjusting for the preintervention scores (covariant). Levene test and normality checks were carried out, and the assumptions met. There was a significant difference in the GAD-7 score reduction (*F*_1,43_=7.307; *P*=.01) between the two arms. Parameter estimates showed that the GAD-7 scores for the intervention group were significantly reduced (β=−3.27; *P*=.01), with an adjusted *R^2^* value of 0.106. There was a significant difference in the PHQ-9 score reduction as well (*F*_1,43_=8.298; *P*=.006) between the intervention and control groups. Parameter estimates showed that the PHQ-9 scores in the intervention group were significantly reduced (β=−4.311; *P*=.006) with an adjusted *R^2^* value of 0.173 (Tables 2 and 3).

**Figure 1:**
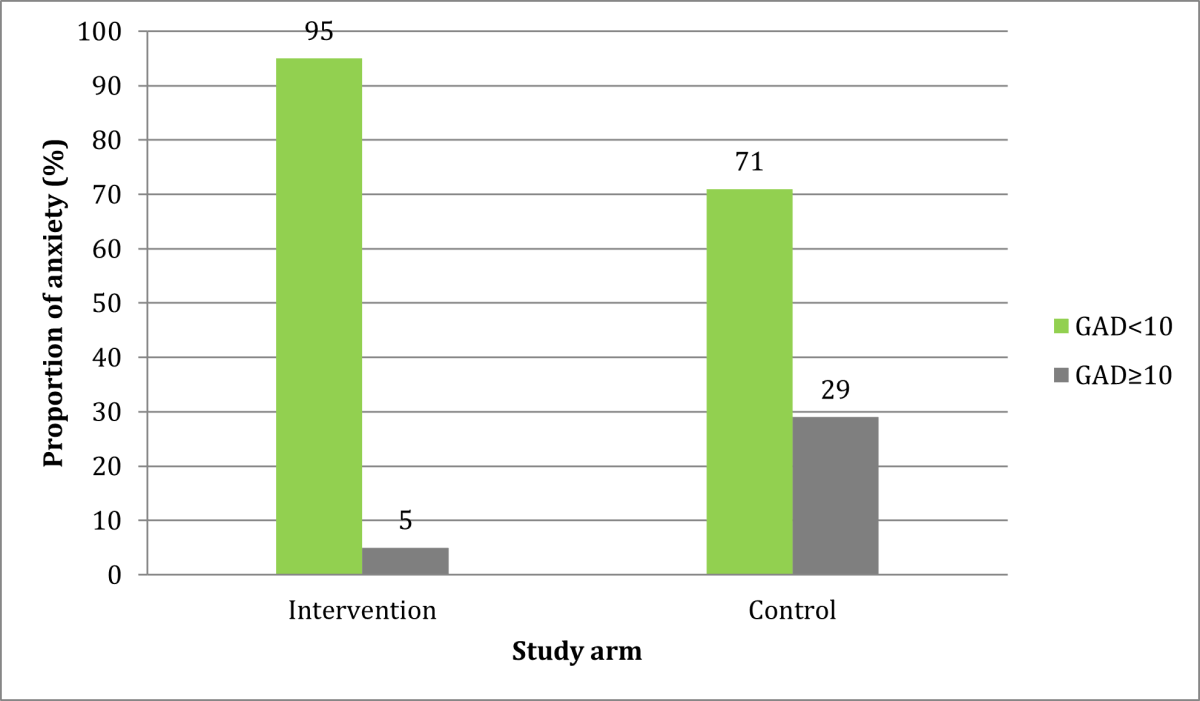
Comparison of the postintervention proportions of anxiety between the two study arms using the chi-square test. GAD-7: Generalized Anxiety Disorder-7 Scale.

**Figure 2:**
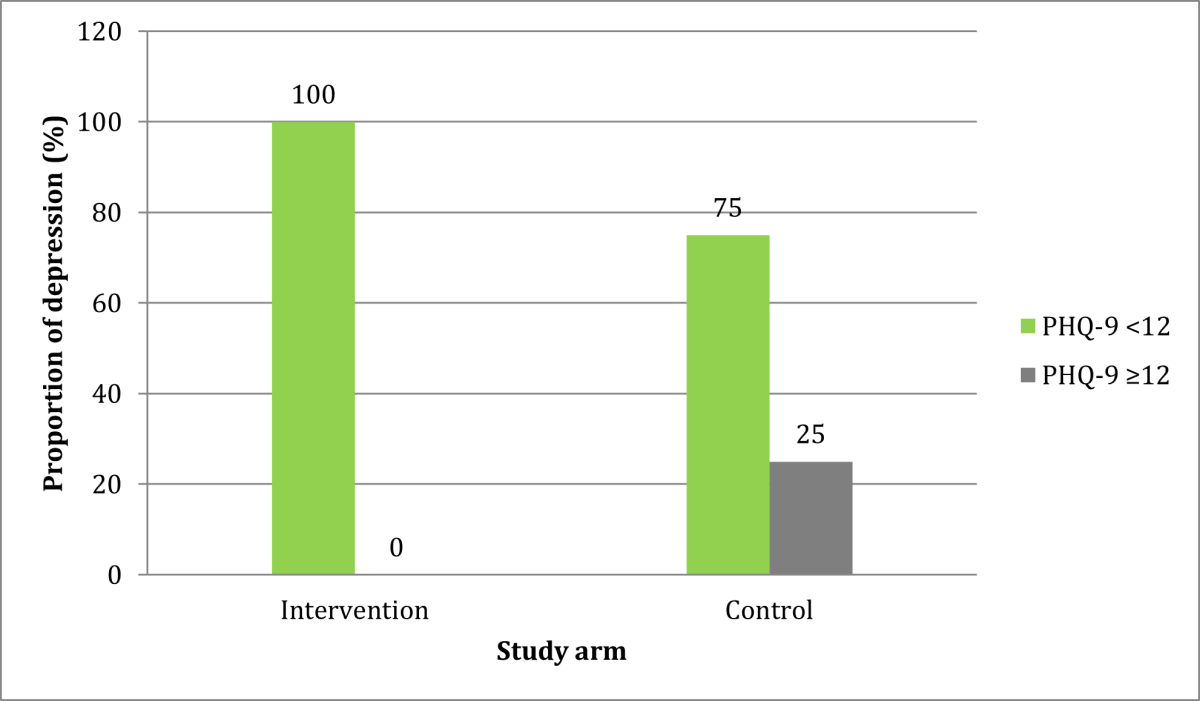
Comparison of the postintervention proportions of depression between the two study arms using the chi-square test. PHQ-9: Patient Questionnaire-9.

Figures 1 and 2 show the comparisons of the proportions of postintervention anxiety and depression, respectively, between the two study arms. Although the levels of anxiety and depression had reduced in both study arms, the proportions of participants with anxiety and depression were significantly lower in the intervention arm than in the control arm (*P*=.049 and *P*=.02, respectively). The difference is more pronounced with regard to the impact of the intervention on depression, as none of the participants in the intervention arm met the cutoff score for depression after they received the intervention.

**Table 1:** Baseline characteristics of the participants in the randomized control trial (N = 46).

Variable		Intervention group (n=22)	Control group (n=24)	Total (N=46)	*P* value^a^	
Age (years), mean (SD)		27.0 (8.72)	29.96 (8.63)	28.51 (8.70)	.14^b^	
**Gender, n (%)**						.07
	Male	2 (9.1)	8 (33.3)	10 (22)		
	Female	20 (90.9)	16 (66.7)	36 (78)		
**Marital status, n (%)**						.15
	Single	15 (68.2)	11 (45.8)	26 (57)		
	Married	7 (31.8)	13 (54.2)	20 (43)		
**Do you have children?** **n (%)**						.22
	Yes	17 (77.3)	14 (58.3)	15 (33)		
	No	5 (22.7)	10 (41.7)	31 (67)		
**Education level, n (%)**						.18
	Secondary school	8 (36.4)	4 (16.7)	12 (26)		
	College and above	14 (63.6)	20 (83.3)	34 (74)		
**Employment status, n (%)**						.38
	Employed	9 (40.9)	14 (58.3)	23 (50)		
	Unemployed	13 (59.1)	10 (41.7)	23 (50)		
**Are you a health care worker?** **n (%)**						.93
	Yes	7 (31.8)	8 (33.3)	15 (33)		
	No	15 (68.2)	16 (66.7)	31 (67)		
**Are you financially stable?** **n (%)**						.75
	Yes	15 (68.2)	18 (75)	33 (72)		
	No	7 (31.8)	6 (25)	13 (28)		
**Have you been diagnosed with chronic illness?** **n (%)**						.22
	Yes	2 (9.1)	0 (0)	2 (4)		
	No	20 (90.9)	24 (100)	44 (96)		
**Did you self-quarantine during the COVID-19 pandemic? n (%)**						.36
	Yes	16 (72.7)	14 (58.3)	30 (65)		
	No	6 (27.3)	10 (41.7)	16 (35)		
**Are you in quarantine now because of the COVID-19 pandemic?** **n (%)**						.25
	Yes	15 (68.2)	12 (50)	27 (59)		
	No	7 (31.8)	12 (50)	19 (41)		
**Disorder, n (%)**						.97
	Anxiety	7 (31.8)	8 (33.3)	15 (33)		
	Depression	5 (22.7)	6 (25)	11 (24)		
	Anxiety and depression	10 (45.5)	10 (41.7)	20 (43)		

^a^Chi-square test unless otherwise specified.

^b^Mann-Whitney test.

**Table 2:** Adjusted postintervention anxiety score (dependent variable) and treatments using analysis of covariance.

Parameter	β	95% CI	*t* test (*df*)	*P* value
Intercept	6.79	2.97-10.62	3.59 (1)	<.001
Pre-GAD-7^a^ score	0.02	−0.24 to 0.27	0.15 (1)	.89
Intervention group	−3.27	−5.71 to −0.83	−2.70 (1)	.01

^a^GAD-7: Generalized Anxiety Disorder-7 scale.

**Table 3:** Adjusted postintervention depression score (dependent variable) and treatments using analysis of covariance.

Parameter	β	95% CI	*t* test (*df*)	*P* value
Intercept	5.25	0.80-9.71	2.38 (1)	.02
Pre-PHQ-9^a^ score	0.23	−0.03 to 0.49	1.77 (1)	.08
Intervention group	−4.31	−7.33 to −1.29	−2.88 (1)	.006

^a^PHQ-9: Patient Health Questionnaire-9.

## Discussion

### Principal Findings

The primary objective of this study was to measure the efficacy of therapist-guided online treatment protocols versus self-help, emailed-delivered therapy in a sample of the Omani community during the COVID-19 pandemic. In our sample of 46 participants, we found that therapist-guided online therapy led to a significant reduction in psychological distress as determined by the participants’ experience of depression or anxiety. This evidence supports previous studies that have found that online therapy is efficient and provides increased access to treatment for people experiencing anxiety and depression [6,7]. The results of this study align with the existing literature with regard to this alternate mode of delivery of psychotherapy services.

With the rapid spread of the COVID-19 pandemic worldwide, social systems have had to adapt to a changed society, characterized with physical distancing, working from home, and increased levels of uncertainty and fear [1,2]. The pandemic has acted as a catalyst for change in the delivery of health care services. The remote delivery of psychological treatment services has been researched; studies conducted before the pandemic have found that the efficacy of remote-based treatment services is as high as that of face-to-face treatment programs [9,10].

The second objective of this study was to comparatively assess the effectiveness, as defined by proportions of anxiety and depression, of therapist-guided online treatment protocols with that of an internet-based, self-help treatment protocol. Overall, improved therapeutic outcomes were noted for both treatment approaches. However, the strength of these associations varied. In both conditions, effect sizes were large, and most participants demonstrated clinically significant improvements as a function of the treatment. Nevertheless, compared to the self-help (control) group, therapist-guided online therapy resulted in a greater reduction of anxiety and depression. These findings support the notion that therapist-guided online therapy and self-help materials are functionally distinct from one another [22].

The randomized allocation ensured balancing baseline characteristics of the study participants in the two arms to control for and monitor factors that could potentially contribute to differential outcomes. Therefore, there were no observed differences in the participants’ expectations of the efficacy for the two treatment approaches. Nevertheless, participants in the control group did show an improvement as a result of the efficacy of the self-help materials, presumably due to the regression of the mean and the natural progression of these conditions with time [22].

In addition to the role of the therapist-client relationship established via an electronic medium, there are other factors that could contribute to the existing results. The reduction in distress levels may have been impacted by the role of the community in times of crisis in a collectivistic society [23], the predicted adjustment to the “new normal” through increased resilience [24] and a decline in pandemic-related information in the media [25].

### Strengths and Limitations

The present study has a number of notable strengths. To the best of our knowledge, this is the first study in the Middle East region to evaluate the efficacy of therapist-guided online therapy services that have been implemented in the region since the onset of the COVID-19 pandemic. Second, treatments were not manualized, thereby allowing therapists to individualize the delivery of each treatment-specific intervention for each participant as they deemed appropriate. Nonetheless, nonmanualized therapy limits the reproducibility of the study.

A limitation of the study is the generalizability of the results. The participants were relatively well-educated and could be classified as “younger adults,” limiting the conclusions with respect to feasibility. In addition, although participation in this study only required basic email skills, not all households in Oman have a broadband internet connection, making our sample less generalizable to the general public. Although it can be argued that most internet users are women [26], it is also worth noting that an overall stigma towards seeking mental health services is evident in Oman [27]. Furthermore, men may be less open to discussing emotional difficulties in Oman and in Eastern culture [28]. Another limitation is that the design of this study did not allow for adequate follow-up of participants, and it is, therefore, unknown whether the treatment effects will be similarly maintained in the long term. Further, the compliance to self-help therapy could not be ascertained fully, and this may limit the interpretation of the findings.

### Conclusions

The findings of this study are consistent with a growing body of the literature demonstrating the effectiveness of online therapy on the psychological impact of COVID-19. Furthermore, the results indicate that therapist support makes a substantial difference in terms of effectiveness of online interventions. Therefore, it can be useful to integrate internet-delivered services with traditional mental health services by using a stepped care model, starting with e-guided self-help and thereafter moving to therapist-guided therapy. Individuals identified to be suitable for online interventions could be directed to therapist-guided programs, whereas those deemed unsuitable can be provided with face-to-face interventions or a hybrid approach. Long-term follow-up may be more valuable and is required to identify the effects of online therapy on anxiety and depression levels. Finally, a more direct investigation of the extent of training and supervision is required to train effective therapists, as well as a plan for further specialized services in Oman. There is a limited number of qualified therapists in Oman, with current services focusing predominantly on a medical model for treatment. Further studies on therapist compatibility and training in evidence-based interventions are needed to make online therapy a more abundant and acceptable option in Oman.

## Appendix

Multimedia Appendix 1 CONSORT-EHEALTH checklist (V 1.6.1).

## Abbreviations

ACT: acceptance and commitment therapy
CBT: cognitive behavioral therapy
GAD-7: General Anxiety Disorder-7
PHQ-9: Patient Health Questionnaire-9
